# Criminal Behaviour Associated with a Novel Mutation in the VPS13A-Gene Causing Chorea-Acanthocytosis

**DOI:** 10.1155/2019/5947416

**Published:** 2019-04-15

**Authors:** Søren Bruno Elmgreen

**Affiliations:** Department of Neurology, Aarhus University Hospital, Nørrebrogade 44, 8000 Aarhus, Denmark

## Abstract

Heralded by obsessive-compulsive disorder and anxiety, chorea-acanthocytosis may initially present in a psychiatric setting. As insidious onset of involuntary movements is commonly precipitated by dopamine blocking agents, this may not prompt further neurological investigation until symptoms progress after withdrawal of the suspected offending drug. Oromandibular dystonia and frontal disinhibition should call for early neurologic evaluation.

## 1. Introduction

Involuntary movements presenting in a psychiatric setting are often precipitated by use of dopamine blocking agents; however, the presence of oromandibular dystonia and frontal disinhibition should call for early neurological investigation. Highlighting the typical neuropsychiatric features of the condition, the first confirmed example of explicitly criminal behaviour in a case of chorea-acanthocytosis is presented, incidentally caused by a novel mutation in the VPS13A-gene.

## 2. Case Presentation

Aged 25, a man of Turkish ancestry was referred for psychiatric evaluation due to re-emergence of obsessive-compulsive behaviour with mysophobia present transiently from the age of 9 till 10. The patient was diagnosed with obsessive-compulsive disorder and symptoms were initially relieved significantly by sertraline which was later augmented by ziprasidone due to psychotic compulsions.

At 28 years of age, management became increasingly problematic as the patient began demonstrating uninhibited behaviour, soliciting and propositioning nurses; furthermore, the patient developed a substantial drug abuse and started home-growing cannabis.

At 29 years of age, the patient robbed a convenience store to pay an outstanding electric bill; he was sentenced to mental observation. During observation the treating psychiatrist noted tics, facial stereotypies, and compulsive coughing. Symptoms were attributed to extrapyramidal side effects of the antipsychotic medication and treatment with ziprasidone was discontinued.

Symptoms, however, progressed slowly during the next six months to encompass complaints of spilling fluids when drinking and accidentally biting cheeks when eating; moreover, the patient noted mild slurring of the speech and developed involuntary movements involving the face and limbs.

At 31 years of age, the patient was referred for neurological evaluation.

The patient's parents were first cousins with no evidence of psychiatric or neurologic diseases; two out of three older siblings were exhibiting obsessions and compulsions resembling those of the patient but had not been evaluated.

On clinical examination, the patient showed dystonic posturing of the hands and feet, and a mild limb chorea most pronounced in the upper extremities. Although there was distal muscle wasting, muscle power was preserved. Tendon reflexes were absent. Sensory examination was normal.

Most remarkably, the patient had marked orofacial dyskinesia with prominent tongue dystonia and tongue protrusion on feeding. The patient exhibited involuntary self-mutilating behaviour with biting of tongue and cheeks and severe bruxism. Speech was moderately dysarthric and accompanied by vocalisations in the form of lip smacking and repeated sniffling.

**Figure 1:**
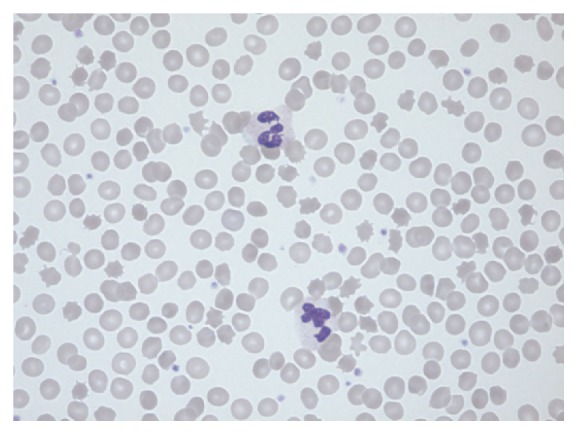
Peripheral blood smear demonstrating the typical appearance of spiked erythrocytes, acanthocytes.

Blood screening revealed elevated creatine phosphokinase to 5164 U/l. Liver enzymes were normal. Copper screening was normal, and no Kayser-Fleischer rings were found. Brain MRI was normal as was cardiac and abdominal ultrasound. Nerve conduction studies showed a sensory axonal polyneuropathy. Repeated peripheral blood smears showed 20-30% acanthocytes (see Figure 1).

Sequencing of the VPS13A-gene revealed a novel homozygous mutation (c.1186del) with subsequent nonsense-mediated RNA-decay (p.Val395Serfs*∗*4) confirming the diagnosis of chorea-acanthocytosis. The patient's parents were both heterozygous for the mutation; the patient's siblings have opted not to be tested.

## 3. Discussion

chorea-acanthocytosis is part of the neuroacanthocytosis syndromes including a range of neurodegenerative disorders associated with the presence of acanthocytes in peripheral blood smears; other disorders included under this umbrella heading are Huntington disease-like 2, panthetonate kinase-associated neurodegeneration, and McLeod syndrome.

Best estimates put the number of cases of chorea-acanthocytosis around 500 to 1,000 worldwide; the most consistently reported mean age of onset is around 30 years of age [1–4]. Patients are affected by a mixed movement disorder with mild limb-onset chorea which progresses to more severe generalized chorea or even parkinsonism [1]. Additionally, some patients develop generalized tonic-clonic seizures possibly secondary to complex focal seizures arising in the mesial temporal lobe [2].

Diagnosis should be suspected in individuals presenting orofacial dystonia, and marked tongue involvement [3]. So-called feeding dystonia, characteristic head drops, and violent trunk spasms are features highly suggestive of chorea-acanthocytosis and self-injurious behaviour is not uncommon [5, 6].

Disease onset is often heralded by psychiatric and behavioural disturbances several years prior to onset of motor symptoms and chorea-acanthocytosis may initially be mistaken for extrapyramidal side effects of dopamine blocking agents [1, 4].

Patients are most often affected by obsessive-compulsive disorder, but also schizophrenia, apathy, anxiety, and depression have been described [1, 4, 7, 8]. Neuropsychological testing often reveals a frontal dysexecutive syndrome, poor judgement, and social disinhibition [4]. The presented case is, to the best of our knowledge, the first verified report of criminal behaviour in a case of chorea-acanthocytosis.

A sensory axonal polyneuropathy can be readily demonstrated by nerve conduction studies and clinical or subclinical myopathy evident by elevated creatine phosphokinase. Peripheral blood smear usually shows 5-50% acanthocytes; however this trait is variable [9, 10]. Ancillary testing should include echocardiography as rare cases of cardiomyopathy have been described [11].

Magnetic resonance imaging of the brain may show atrophy of the putamen and the caudate head, increased signal in the basal ganglia on T2-weighted sequences, and evidence of iron deposition on susceptibility-weighted sequences [3, 9, 12–14].

Diagnosis has traditionally been made by demonstrating reduced or absent chorein by means of Western blot on erythrocytes [4]. With the advances in genetics, sequencing of the entire VPS13A-gene can now be undertaken at reasonable cost [15].

Management of chorea-acanthocytosis is symptomatic. Anticholinergics, dopamine-depleting drugs, and injections with botulinum toxin may be useful in controlling motor symptoms [9, 16]. Moreover, there have been several reports of benefit from deep brain stimulation of the internal part of the globus pallidus [17, 18]. A gastrostomy may be needed to maintain nutritional status while physiotherapy and occupational therapy may help patients to maintain functions of daily living. In time, however, the relentless progression results in major disability which almost inevitable leads to dependency.
